# Clinical Pharmacology of Chemotherapy Agents in Older People with Cancer

**DOI:** 10.1155/2011/628670

**Published:** 2011-08-10

**Authors:** Xiaoye He, Stephen J. Clarke, Andrew J. McLachlan

**Affiliations:** ^1^Department of Geriatrics, Zhongshan Hospital, Fudan University, Shanghai 200032, China; ^2^Faculty of Pharmacy, University of Sydney, NSW 2006, Australia; ^3^Sydney Medical School and Royal North Shore Hospital, University of Sydney, NSW 2006, Australia; ^4^Centre for Education and Research on Ageing, Concord Hospital, NSW 2139, Australia

## Abstract

Populations around the world are aging, and the associated increase in cancer incidence has led to the recognition of the importance of geriatric oncology. Chronological age is a poor determinant of pharmacological response to cancer chemotherapy agents. Age-associated changes in physiology and organ function have a significant impact on the clinical pharmacology of cancer chemotherapy agents used in cancer treatment. Altered response to medicines in older people is a consequence of changes in body composition, organ function, concomitant pathophysiology, multiple medications, genetic determinants of drug response, and patient's clinical status. These issues highlight the need to individualize the management of cancer in the older people with consideration of age-related changes in the clinical pharmacology of cancer drugs, analgesics, and adjunctive therapies.

## 1. Introduction

The continued growth in the proportion of older people in many developed countries has resulted in a large number of older adults with cancer. Cancer is now the second most common cause of death, and approximately half of all cancers occur in the population aged 65 years and older [1]. There is an increasing number of older patients who require the management of cancer. Understanding and defining the optimum use of cancer chemotherapy agents in older cancer patients in the adjuvant, curative, and palliative settings is a very pressing and timely issue in oncology. However, until recently, the area of geriatric oncology has been relatively ignored and there has been less research undertaken than in other areas of oncology which has resulted in a lack of evidence-based guidelines for the treatment of older cancer patients. A significant challenge in providing cancer care to older people is that aging is an individualized and heterogeneous process with variable effects on the clinical pharmacology of drugs used in the management of cancer. This has important implications for choosing the optimal drug regimen, selecting a safe and effective initial, dose and undertaking appropriate monitoring strategies in managing older people with cancer. 

The aim of this paper is to identify and discuss the factors which affect the clinical pharmacology of chemotherapy agents in older people with cancer and the medical management of these patients.

## 2. Factors Affected the Clinical Pharmacology of Chemotherapy in Older Cancer Patients

### 2.1. Age-Related Changes in Clinical Pharmacology

#### 2.1.1. Pharmacokinetics

Studies investigating age-related changes in the pharmacokinetics of cancer chemotherapy agents have provided conflicting evidence [2]. While some studies report age-related differences in the pharmacokinetics of cancer chemotherapy agents, most studies have reported no significant differences based on patient chronological age *per se*, or only minor differences in the pharmacokinetics of chemotherapy agents in older people. However, many of the older patients included in these studies have been selected on the basis of good performance status and normal organ function and thus may not be representative of patients treated in routine practice. Changes observed in the pharmacokinetics of drugs in older people (when compared to younger people) result from age-related changes in physiology and organ function and/or comorbid disease [3].

Pharmacokinetic assessments in patients should incorporate consideration of factors affecting drug absorption, distribution, metabolism, and excretion. 

Drug absorption can be affected by age-related changes in the physiology of the gastrointestinal tract such as decreased splanchnic circulation, reduced gastric motility and secretion, and reduction in the absorptive surface area for drugs [4, 5]. However, there are relatively few examples of drugs affected by these changes which result in clinically relevant alterations in the extent of drug absorption. Typically the rate of absorption may be reduced in older people but the extent of absorption (associated with bioavailability) remains largely unaffected [3] in older patients. Age-associated changes in gastrointestinal physiology and the potential effects on drug absorption may come into sharper focus with the trend for increased use of oral chemotherapeutic drugs. This is an important area for future investigation.

Aging is associated with changes in body composition, including a reduction in total body water, protein stores, and lean body mass with an increase in proportion of body fat [6]. Decreased intracellular water leads to a reduced volume of distribution (V) for hydrophilic drugs that primarily distribute to body water, while the relative increase in adipose tissue in older people can result in an increase in the volume of distribution of lipid-soluble drugs. This may result in a reduction in the maximum concentration achieved after a dose and prolongation of the terminal half-life of an agent in older people when compared to younger patients [7]. The reduction in serum albumin concentration in older adults results in an increase in the unbound fraction of some drugs, which may have important implications [8] for the distribution of drugs bound to albumin. Studies had shown that low serum albumin concentrations in malnourished older patients with advanced cancer resulted in a low clearance of highly albumin-bound drugs which, in turn, caused increased free drug concentration and contributed to unexpected toxicity [9]. 

Clearance, a measure of the efficiency of drug elimination, is the most important pharmacokinetic variable as it is the main determinant of maintenance dose rate for multiple dosing drug regimens and of drug exposure (i.e., area-under-the-concentration-time curve, AUC) after a single dose. Drug metabolism has the greatest impact on clearance for most drugs (and in turn, exposure) and is therefore likely to significantly influence a patient's beneficial and adverse response at a given dose [10]. The liver is the principal site of drug metabolism. Age-related changes in the liver include decreased liver weight, reduced hepatic blood flow, and reduced amount and activity of cytochrome P450 enzymes, in association with an overall reduction in the metabolic capacity of the liver in older people [4, 5]. Many chemotherapy agents, including the taxanes (paclitaxel and docetaxel), cyclophosphamide, and vinca alkaloid drugs (vincristine, vinblastine, and vinorelbine), and molecular targeted drugs are metabolized by cytochrome P450 enzymes, especially CYP3A4, and predominantly undergo hepatic metabolism and clearance [7]. There is clear evidence that the liver sinusoidal endothelial cells in the aging liver undergo pseudocapillarization such that the sieving function of liver is significantly reduced with age [11]. This age-related change in the liver has implications for highly protein bound drugs and large macromolecules or protein-based therapeutic agents (such as liposomal therapies, e.g., liposomal doxorubicin) with the potential for a reduction in hepatic clearance due to reduced access to the hepatocytes. Biliary excretion is a key hepatic elimination pathway for numerous drugs and their metabolites. While this is a major pathway of elimination of commonly used drugs such as the anthracyclines (doxorubicin, epirubicin, and daunorubicin), it appears to be unaffected by the age of the patient and the associated changes in physiology [12]. 

There is increasing recognition that inflammation associated with advanced cancer and frailty in older people has a significant effect on the regulation of drug metabolising enzymes and transporters [13, 14]. It has been demonstrated in both clinical and preclinical studies that elevated plasma concentrations of inflammatory proteins are associated with reduced hepatic drug clearance and increased toxicity from chemotherapy [15]. Inflammation has the potential to downregulate drug metabolism and transporter pathways further complicating the impact of advanced cancer and frailty on the clinical pharmacology of cancer chemotherapy agents [16–18]. Given the impact of inflammation on drug metabolism, it is, therefore, not unreasonable to hypothesize that the level of inflammatory markers in blood might correlate with variations seen in the metabolism of anticancer drugs. However, this relationship should be evaluated in prospective trials incorporating pharmacokinetic analyses of cytotoxic drugs.

Many drugs and metabolites are renally eliminated from the body. Renal clearance is the result of glomerular filtration, renal secretion (mediated by transporters), and tubular reabsorption. A reduction in glomerular filtration rate (GFR) is almost universal with age, and it has been reported that there is a decrease in the GFR of approximately 1 mL/minute for every year over the age of 40 years [19]. The kidneys' ability to appropriately concentrate or dilute urine and excrete water and electrolytes is also impaired with aging [5, 20]. This decline in renal function may be associated with an increased risk of toxicity for renally excreted cytotoxic agents and their metabolites, such as platinum compounds, alkylating agents, capecitabine, purine analogues, antimetabolites, camptothecins, and etoposide [7]. It is recommended to estimate the creatinine clearance (CrCl, as a marker of a person's renal function) with the formula of Cockcroft and Gault [21] in all patients with age of 50 years or more to guide the drug and dosage selection [3, 22, 23]. While there is now a range of equations which have been used to estimate a person's GFR based on serum creatinine concentration, the CrCL remains the best and most reliable metric to estimate renal function for the determination of drug dose regimens for renally excreted medicines [3, 21, 23].

#### 2.1.2. Pharmacodynamics

Most of the age-related differences in drug response observed in older cancer patients are in the realm of pharmacodynamics and manifest as decrements in end-organ function [3]. Age-related changes in effector system function, organ function, and impaired homeostatic control result in age-related changes in pharmacodynamics. End-organ response is affected by physiological changes that occur with increasing age in the absence of pathology [23] or in the context of concomitant multiple pathophysiological changes. For example, the diminished homeostatic reserve in older people [3], especially those that are frail, leads to a greater impact of haematological toxicities from cancer chemotherapy agents.

The pharmacodynamic changes of aging may affect both the efficacy and the toxicity of many antineoplastic agents. The increased prevalence of MDR-1 in acute myeloid leukemia [24], increased resistance to apoptosis in follicular lymphoma [25], increased adhesion of neoplastic cells to stroma in multiple myeloma [26], decreased tumour growth fraction [27], tumour cell anoxia, and abnormal chemotherapy targets [28] are all examples of age-associated changes in pharmacodynamics that impact the efficacy of chemotherapy agents in older cancer patients.

The impaired homeostatic control associated with aging also increases the risk of short- and medium-term complications of cancer chemotherapy, including myelosuppression [29], acute cardiomyopathy [30], peripheral and central neuropathy [31] and mucositis [32], as well as long-term complications [33–36] such as chronic cardiomyopathy from anthracyclines, increased incidence of myelodysplasia (MDS), and acute myeloid leukemia (AML).

### 2.2. Comorbidity

The occurrence of comorbid medical conditions such as diabetes, heart disease, hypertension, arthritis, and lung disease affects many older patients [37]. The likelihood of an older person experiencing a chronic illness increases rapidly with age. In cancer patients, comorbidity limits treatment options, negatively affects treatment tolerance, and influences the presence and severity of symptoms and other complications [38]. For example, more attention should be paid to patients with ischemic heart disease when they are treated with fluoropyrimidines as a previous study has demonstrated that patients with a history of cardiac disease, particularly coronary artery disease, were significantly more susceptible to 5-FU cardiotoxicity when compared to patients without this medical history [39]. Patients with impaired lung function will face increased risk of pulmonary toxicity from bleomycin. Giving cisplatin to patients with poor cardiac function will increase the risk of the onset of heart failure due to the requirement for prehydration with large volumes of fluids. Comorbidity is also associated with poorer survival, increasing both cancer and noncancer related mortality [40, 41]. The treatment of comorbid conditions during cancer treatment may result in an increased likelihood of drug interactions. In addition, cancer treatments, including chemotherapy, radiation therapy, and other supportive care drugs, may exacerbate comorbid conditions [23].

### 2.3. Polypharmacy

The obvious consequence of comorbidity is the concurrent use of multiple medications. Of all the factors that are most consistently associated with adverse drug reactions, inappropriate or unnecessary polypharmacy has been considered the most important. Using multivariate analysis, studies have demonstrated that the principal contributor to adverse drug reactions in older people is inappropriate polypharmacy [23]. The harms associated with polypharmacy include increased risks of adverse drug reactions, drug interactions, increased healthcare costs, and errors in patient adherence to therapy [23]. Older age, comorbidity, recent hospitalization, female gender, depression, number of treating doctors, and practitioner characteristics are the main risk factors for polypharmacy [42]. Some medicines place older people at a significantly higher risk of adverse effects and serious drug interactions [42]. The Beers Criteria provide an important starting point to improve prescribing by limiting the use of medicines that pose a high risk of adverse effects in older people [42, 43].

## 3. Medical Management in Older People with Cancer

### 3.1. Comprehensive Geriatric Assessment

A comprehensive geriatric assessment (CGA) approach, including identification of frailty, can assist oncologists in identifying older patients who are more likely to develop severe toxicity during cancer treatment [44]. Components of the GCA, which involves a multidimensional evaluation to determine the overall health status of an older adult, have been used effectively to guide treatment planning and decision making in older adults with cancer. The CGA determines which patients may benefit from and tolerate standard cancer treatment including identifying those most likely to derive benefit from a palliative treatment approach [45].

Although the CGA is a useful tool in the assessment of older people with cancer, this approach is time-consuming and is only indicated in selected patients [46, 47]. Some clinical oncologists have used an abbreviated tool such as the VES-13 to screen patients who should go on to a full CGA [48]. In addition to this, the National Comprehensive Cancer Network (NCCN) has developed useful guidelines for pretreatment screening to determine which patients, such as those aged 70 years or more and whose hemoglobin levels are under 12 mg/dL, who may need a more comprehensive assessment [49].

### 3.2. Treatment Strategies

Medical oncologists have gradually started to appreciate that the approach to cancer chemotherapy that is adopted for otherwise healthy adults cannot automatically be applied to older patients with age-related changes in physiology, reduced homeostatic reserve, and comorbid medical problems. Numerous questions need to be addressed prior to commencing chemotherapy in older patients including which anticancer agents should we choose in each particular circumstance? Should we use single agents or combination chemotherapy? Should we use oral or intravenous administration? These issues must be taken into consideration by the oncologists when determining treatment strategies in older cancer patients [50]. Chemotherapy regimens that equally optimise efficiency, tolerance, and compliance should be preferred in older cancer patients. However, the strategy of using and applying the best clinical evidence to guide treatment selection is a considerable challenge as older patients are often excluded from clinical trials.

### 3.3. Efficiency and Toxicity

Although standard treatment regimens may be safe and effective in older adults, the treatment of cancer in older patients requires an individualized approach. As discussed, older patients are more prone to toxicity from chemotherapeutic agents, due to age-related changes in both pharmacokinetics and pharmacodynamics [51]. The common treatment toxicities of particular concern when caring for older adults with cancer are myelosuppression, mucositis, cardiotoxicity, neurotoxicity, and musculoskeletal side effects [52].

## 4. Frailty

Frail older people are defined as those with an excess reduction in lean body mass and mobility, poor tolerance to therapy and fatigue, presence of geriatric syndromes, and/or the presence of multiple comorbidities [53]. Frailty reflects a common view of aging, indicating a critically exhausted functional reserve [54]. The recognition of frailty is particularly useful in planning cancer chemotherapy in older individuals. It may allow the practitioner to identify patients who are most at risk of chemotherapy-induced adverse outcomes and to implement a strategy to monitor and prevent these complications [53]. The frail older cancer patient needs a different approach to chemotherapy prescription. Sarcopenia, inflammation, and poor nutritional status are associated with frailty in older people [52, 54, 55]. The factors have the potential to significantly affect the clinical pharmacology of cancer chemotherapy agents. Simple pharmacological palliation of symptoms, rather than administration of potentially toxic chemotherapy, may be the preferred option in frail older people; however, the rationale for this option may need careful, sympathetic, and lengthy explanation to the patient and their relatives. Relatively less toxic single agent therapies may be the best choice in many solid tumour types, but may not be effective in rapidly growing tumours such as high-grade non-Hodgkin lymphomas or acute myeloid leukaemia [50].

## 5. Summary and Outlook for the Future

Older people represent an increasing proportion of the population and are a heterogeneous patient group at high risk for developing cancer. Age-related physiological changes may have a considerable impact on the pharmacokinetic and pharmacodynamic properties of cancer chemotherapy agents. For anticancer drugs, which have a low therapeutic index even in optimal circumstances, pharmacological changes can result in dramatic consequences, such as excessive increases in drug concentrations that produce severe and even life-threatening toxicities when standard dosing regimens are employed. In general, chronological age *per se* is not a contraindication to receiving cancer chemotherapy [56]. Comorbidity and poor functional status, which may be present in a significant number of the older patient population, are the main limiting factors. The key strategy is to focus on the clinical pharmacology of cancer chemotherapy agents and to individualises treatments which can be achieved by understanding the nature and extent of age-related changes in physiology and organ function. Older cancer patients, especially those with comorbidities, have been historically omitted from clinical trials resulting in study populations that are selected for their fitness and thus not representative of typical older cancer patients. It still remains a challenge to tailor and deliver the most beneficial treatments for those over the age of 65 years, taking into account comorbidities and physiologic reserves. Fortunately, there are clinical trials within various cooperative groups directed toward the development of effective and safe treatment strategies for the older people with cancer. These considerations will have important implications for training the next generation of healthcare professionals to meet the future needs [57] of older people living with cancer.
